# Meta-analysis of the prevalence of livestock diseases in North Eastern Region of India

**DOI:** 10.14202/vetworld.2020.80-91

**Published:** 2020-01-11

**Authors:** Nagendra Nath Barman, Sharanagouda S. Patil, Rashmi Kurli, Pankaj Deka, Durlav Prasad Bora, Giti Deka, Kempanahalli M. Ranjitha, Channappagowda Shivaranjini, Parimal Roy, Kuralayanapalya P. Suresh

**Affiliations:** 1 Department of Veterinary Microbiology, College of Veterinary Science, Assam Agriculture University, Guwahati, Assam, India; 2 Department of Virology, ICAR-National Institute of Veterinary Epidemiology and Disease Informatics, Bengaluru, Karnataka, India; 3 Department of Spatial Epidemiology, ICAR-National Institute of Veterinary Epidemiology and Disease Informatics, Bengaluru, Karnataka, India; 4 Director, ICAR-National Institute of Veterinary Epidemiology and Disease Informatics, Bengaluru, Karnataka, India

**Keywords:** babesiosis, brucellosis, classical swine fever, foot-and-mouth disease, forest plot, livestock, meta-analysis, North Eastern regions, Peste des petits ruminants, *Porcine circovirus*, porcine cysticercosis, porcine reproductive and respiratory syndrome, prevalence, seroprevalence, theileriosis

## Abstract

**Aim:**

The study aimed to determine the overall prevalence of livestock diseases in North Eastern Region (NER) of India, through a systematic review and meta-analysis of published data.

**Materials and Methods:**

The articles used for the study were retrieved from PubMed, J-Gate Plus, Indian Journals, and Google scholar, R open-source scripting software 3.4.3. Metafor, Meta. The Chi-square test was conducted to assess for the heterogeneity, forest plot (confidence interval [CI] plot) is a method utilized to present the results of meta-analysis, displaying effect estimate and their CIs for each study were used for searching and retrieval of livestock diseases prevalence data in India using a search strategy combining keywords and related database-specific subject terms from 2008 to 2017 in English only.

**Results:**

The prevalence of various livestock diseases are foot-and-mouth disease (21%), bluetongue (28%), brucellosis in bovine (17%), brucellosis in caprine (2%), brucellosis in porcine (18%), brucellosis in sheep and goat (3%), babesiosis (6%), theileriosis (26%), porcine reproductive and respiratory syndrome (1%), porcine cysticercosis (6%), classical swine fever (31%), *Porcine circovirus* (43%), and Peste des petits ruminants (15%). This information helps policymakers to take appropriate measures to reduce the disease burden.

**Conclusion:**

This study indicates that the overall prevalence of various livestock diseases in NER of India.

## Introduction

The term “livestock” is nebulous and livestock refers to any breed or population of animals kept by humans for a useful, commercial purpose. India is the world’s highest livestock owner at about 512.05 million [1]. Livestock plays an important role in the Indian economy. About 20.5 million people depend on livestock for their livelihood. Livestock contributed 16% to the income of small farm households as against an average of 14% for all rural households. Livestock provides a livelihood to two-third of the rural community. It also provides employment to about 8.8% of the population in India. India has vast livestock resources. The livestock sector contributes 0.15% point to the gross domestic product (GDP) growth and the share of livestock to the GDP was recorded at 4.29% and 25.6% of total agriculture GDP [2].

The North Eastern Region (NER) of India comprising the states of Assam, Arunachal Pradesh, Manipur, Meghalaya, Mizoram, Nagaland, Sikkim, and Tripura occupies about 7% of total land area and 4% of the total population of the country. Agriculture is the prime source of livelihood for the majority (85%) of the rural population in the NER of India, the consumption of meat is relatively higher in this region, and that of milk and milk products is lower. Coupled with the traditional meat-eating habit, increasing per capita income, urbanization, and changes in lifestyle, the region is deficit in the production of livestock products. Some states in the region depend on inter-state trade in livestock to meet the domestic demand [3]. Livestock disease affects the economy, animal welfare, the environment, and public health. Animals are susceptible to a number of diseases and conditions that may affect their health. Some of the important livestock diseases in NER are foot-and-mouth disease, bluetongue, brucellosis in bovine, porcine, sheep and goat, babesiosis, theileriosis, porcine reproductive and respiratory syndrome **(**PRRS), porcine cysticercosis, classical swine fever (CSF), *Porcine circovirus* (PCV), and Peste des petits ruminants (PPR). Where the disease condition is serious, governments impose regulations on import and export, on the movement of stock, quarantine restrictions, and the reporting of suspected cases. Vaccines are available against certain viral diseases of livestock and antibiotics are rampantly used against bacterial diseases which require regular surveillance [4]. However, the growth of the livestock sector has been slower in NER than at the national level which needs to be improved. Meta-analysis is a quantitative, formal, and epidemiological study design used to systematically assess the previous research studies to derive conclusions about that body of research. Outcomes from a meta-analysis may include a more precise estimate of the effect of treatment or risk factor for disease, or other outcomes, than any individual study contributing to the pooled analysis [5]. In recent past years, the prevalence of brucellosis, CSF, bovine tuberculosis, and bovine viral diarrhea virus was reported by different researchers in different countries in the world [7,6-22]. However, the prevalence of livestock diseases in the NER of India has not been studied systematically, and therefore, the prevalence status of the diseases is largely unknown at the country level.

This study aimed to systematically review the existing literature and provides a standard estimate of the prevalence of various livestock diseases in NER in India. This would pave the way for epidemiological modeling, which would help to formulate and evaluate control strategies in the distant future.

## Materials and Methods

### Ethical approval

Ethical approval was not required for this study.

### Study strategy

Literatures were collected from the period 2008 to 2017 using various search engines such as PubMed, J-Gate Plus, Indian Journals, and Google scholar. The search was made using the terms such as babesiosis, theileriosis, foot-and-mouth disease, brucellosis, PRRS, porcine cysticercosis, PCV, PPR, and CSF in North East Region. Manual searches on citations retrieved from original studies and review articles were performed. The search was restricted only to studies published in English language or any Indian Languages.

### Study selection

All the search results were limited to cross-sectional, observational, non-randomized, case-control studies, etc., conducted on the animal population. The studies have to meet the following criteria for inclusion, (i) they have to report the number of positive samples for the particular livestock diseases, (ii) number of animals that have been tested, (iii) year of surveillance or year of study conducted, and (iv) studies with standard confirmatory test were included. Studies were excluded if the number of positive samples was not reported either in frequency or proportion, studies such as review articles were also excluded from the study.

### Data extraction

Full articles were collected and examined; two independent reviewers extracted the attributes or characteristics of each included study in a pre-defined data extraction format. This included year of publication, first author, state, total number of samples/sample size, total positive samples for the livestock diseases, and method used for confirmation of it. Any discrepancy in data extraction was resolved through discussion and consensus.

### Analytical approach

The meta-analysis of the prevalence of livestock diseases in the North East Region of India was conducted using the R open-source scripting software 3.4.3. The R packages used for meta-analysis were Metafor and Meta. A total of 21 studies were included from various regions of the country.

The Chi-square test was conducted to assess for heterogeneity. It was evaluated using tau (τ^2^) value and its level of significance [23]. Results on meta-analysis for the random effect model were used if the heterogeneity between the studies was found to be significant and higher τ^2^. I^2^ statistic, a measure of heterogeneity, indicates the percentage of variance between different studies. If the I^2^ statistic indicates considerable heterogeneity, we combine the summary measures across the studies using a random effect model that assumed that the included study represents a sample from a larger population.

### Strategy adopted for addressing heterogeneity

Numbers of options were used in the present study to address heterogeneity.

1. Check again that data are correct: Errors in unit of analysis, proportion, or prevalence present study may lead to severe heterogeneity because of incorrect extraction
2. Explore heterogeneity: It is our interest to determine the cause of heterogeneity if present among the different studies. We have explored for the presence of heterogeneity by conducting subgroup analysis
3. Performing a random effects model: Fixed effect meta-analysis ignores heterogeneity. Pooled effect estimate from a fixed effect meta-analysis is normally interpreted as being best estimate or prevalence. However, the presence of heterogeneity suggests that there may not be a signal population estimate but a distribution of the number of population effects. Thus, using fixed effect model may be erroneous and random effect model is used to incorporate heterogeneity among the studies
4. Exclude studies: If there is a presence of one or two outline studies (Large tau^2^), we have performed exclusion of these studies from the analysis as they introduce unreliable and bias in the results.

### Forest plot

Forest plot (confidence interval [CI] plot) is a method utilized to present the results of meta-analysis, displaying effect estimate and their CIs for each study. Each study is represented by a square at a point estimate of effect and a horizontal line extending either side of the block depicts a 95% CI.

The area of the block is proportional to the weight assigned to that study in the meta-analysis. Forest plots may include the results if the overall effect from a meta-analysis, normally at the bottom of the graph, and often using a diamond to distinguish from the individual studies.

### Sensitivity analysis

Sensitivity analysis is also been used to examine the effects of studies identified as being atypical concerning conduct or result, or being highly influential in the analysis. This sensitivity is mainly used to explore sources of heterogeneity in the body of the research. In the present study, sensitivity analysis has been employed to detect the influential study by each time omitting one of the studies.

### Stratified analysis

Stratified analysis has been frequently used to reduce the heterogeneity. This approach will reduce but not eliminate heterogeneity. In the present study, stratification is applied for grouping different type of samples such as clinical samples and samples from a healthy animal.

### Assessment of heterogeneity

The heterogeneity was assessed in the present study by conducting meta-analysis. The presence of heterogeneity was indicated by the value of τ^2^ and significance is assessed by p-value by Chi-square test.

## Results

### Prevalence of babesiosis

**Figure-1:**
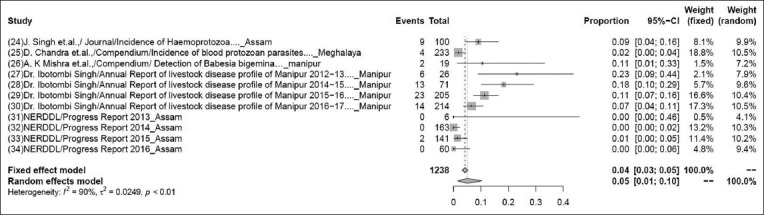
Forest plot of studies on the prevalence of babesiosis.

The pooled prevalence rate of babesiosis in 1238 samples from 11 studies was 6% (95% CI: 3-12%), τ^2^=1.0699; p<0.01** (Figure-1). Although some variations are seen among various studies, the heterogeneity is significant; hence, the random effect model was chosen [24-34].

### Prevalence of bluetongue

**Figure-2:**
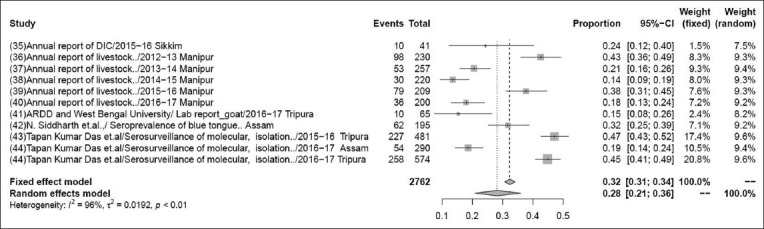
Forest plot of studies on the prevalence of bluetongue.

The pooled prevalence rate of bluetongue in 2762 samples from 11 studies was 28% (95% CI: 21-36%), τ^2^=0.3829; p<0.01** (Figure-2). Although some variations are seen among various studies, the heterogeneity is significant; hence, the random effect model was chosen [35-44].

### Prevalence of foot-and-mouth disease

**Figure-3:**
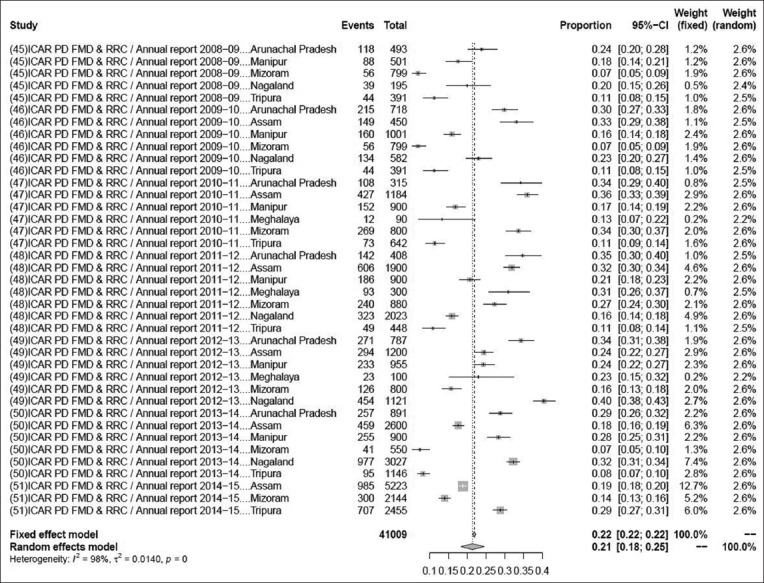
Forest plot of studies on the prevalence of foot-and-mouth disease.

The pooled prevalence rate of foot-and-mouth disease in 41,009 samples from 39 studies was 21% (95% CI: 18-24%), τ^2^=0.3681; p=0.00** (Figure-3). Although some variations are seen among various studies, the heterogeneity is significant; hence, the random effect model was chosen [45-51].

### Prevalence of PPR

**Figure-4:**
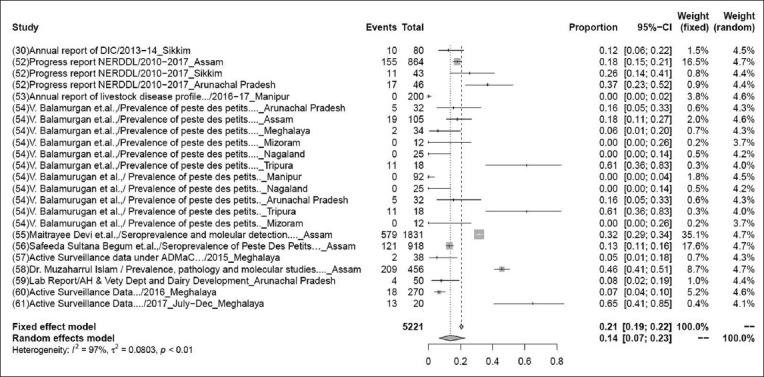
Forest plot of studies on the prevalence of Peste des Petits Ruminants.

The pooled prevalence rate of PPR in 5221 samples from 23 studies was 15% (95% CI: 9-25%), τ^2^=1.6098; p<0.01** (Figure-4). Although some variations are seen among various studies, the heterogeneity is significant; hence, the random effect model was chosen [30,52-61].

### Prevalence of porcine cysticercosis

**Figure-5:**
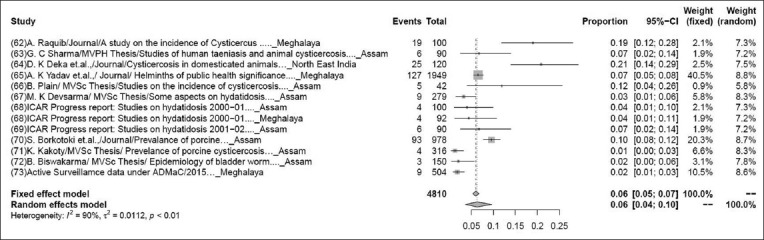
Forest plot of studies on the prevalence of porcine cysticercosis.

The pooled prevalence rate of porcine cysticercosis in 4810 samples from 13 studies was 6% (95% CI: 4-9%), τ^2^=0.7530; p<0.01** (Figure-5). Although some variations are seen among various studies, the heterogeneity is significant; hence, the random effect model was chosen [62-73].

### Prevalence of theileriosis

**Figure-6:**
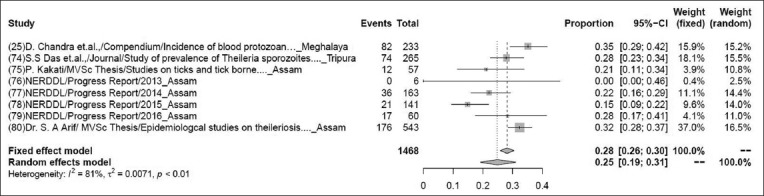
Forest plot of studies on the prevalence of theileriosis.

The pooled prevalence rate of theileriosis in 1468 samples from eight studies was 26% (95% CI: 21-32%), τ^2^=0.1140; p<0.01** (Figure-6). Although some variations are seen among various studies, the heterogeneity is significant; hence, the random effect model was chosen [25,74-80].

### Prevalence of PRRS

**Figure-7:**
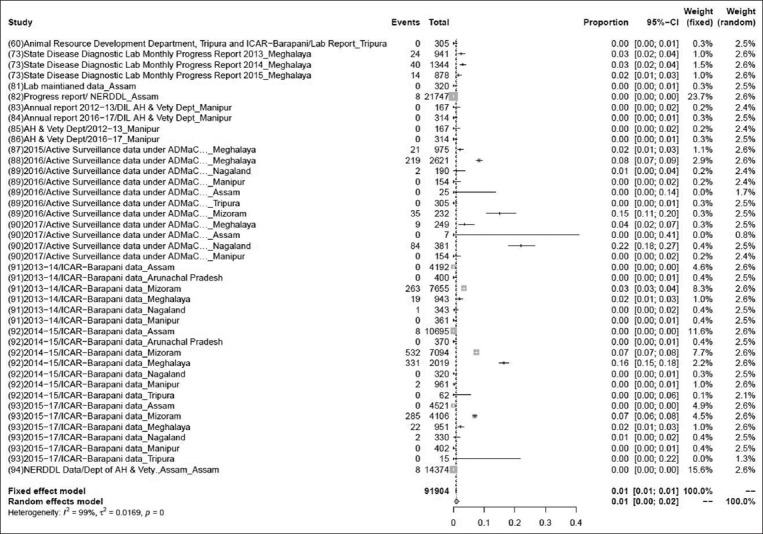
Forest plot of studies on the prevalence of porcine reproductive and respiratory syndrome.

The pooled prevalence rate of PRRS in 91,904 samples from 41 studies was 1% (95% CI: 0-2%), τ^2^=3.5224; p<0.01** (Figure-7). Although some variations are seen among various studies, the heterogeneity is significant; hence, the random effect model was chosen [60,73,81-94].

### Prevalence of brucellosis in bovines

**Figure-8:**
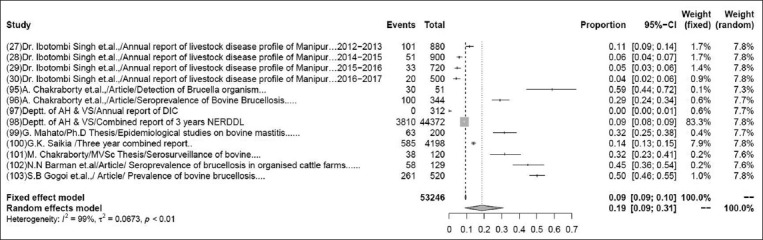
Forest plot of studies on the prevalence of bovine brucellosis.

The pooled prevalence rate of bovine brucellosis in 54,056 samples from 14 studies was 17% (95% CI: 8-29%), τ^2^=0.0654; p<0.01** (Figure-8). Although some variations are seen among various studies, the heterogeneity is significant; hence, the random effect model was chosen [27-30,95-104].

### Prevalence of brucellosis in caprine

**Figure-9:**
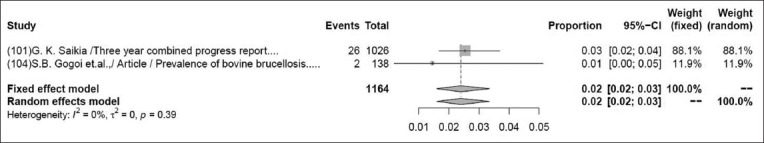
Forest plot of studies on the prevalence of brucellosis in caprine.

The pooled prevalence rate of caprine brucellosis in 1164 samples from two studies was 2% (95% CI: 2-4%), τ^2^=0; p=0.44 (Figure-9). Although some variations are seen among various studies, the heterogeneity is significant; hence, the random effect model was chosen [101,104].

### Prevalence of brucellosis in porcine

**Figure-10:**
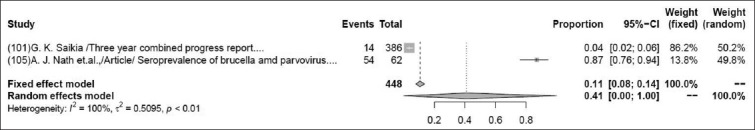
Forest plot of studies on the prevalence of brucellosis in porcine.

The pooled prevalence rate of porcine brucellosis in 448 samples from two studies was 33% (95% CI: 0-99%), τ^2^=13.36; p<0.01** (Figure-10). Although some variations are seen among various studies, the heterogeneity is significant; hence, the random effect model was chosen [101,105].

### Prevalence of brucellosis in sheep and goat

**Figure-11:**
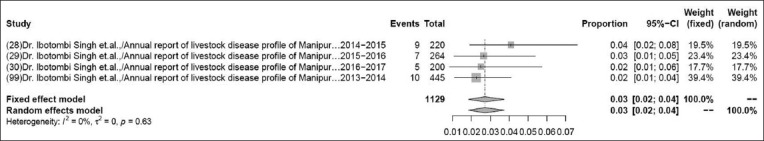
Forest plot of studies on the prevalence of brucellosis in sheep and goat.

The pooled prevalence rate of sheep and goat brucellosis in 1129 samples from four studies was 3% (95% CI: 2-4%), τ^2^=0; p=0.59 (Figure-11). Although some variations are seen among various studies, the heterogeneity is significant; hence, the random effect model was chosen [28-30,99].

### Prevalence of PCV

**Figure-12:**
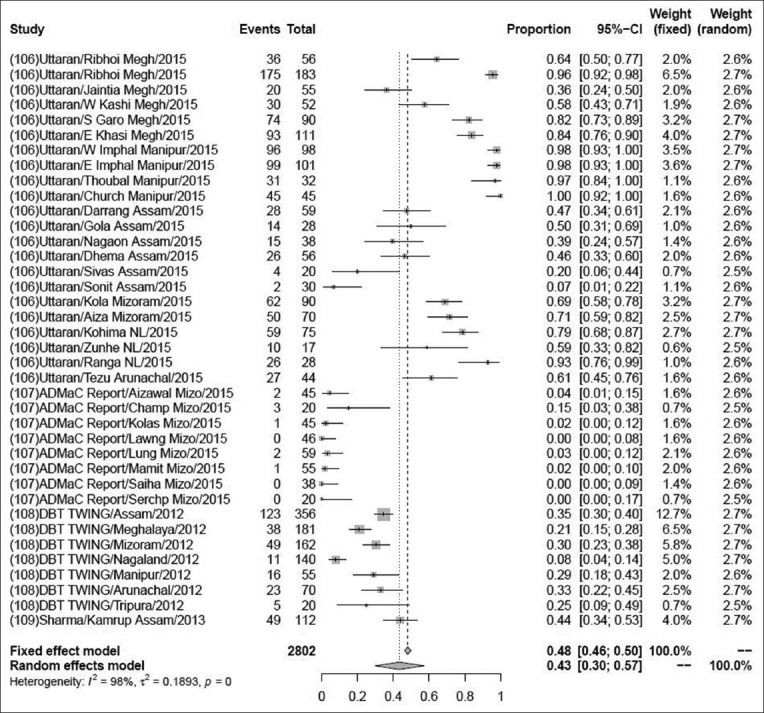
Forest plot of studies on the prevalence of *Porcine circovirus*.

The pooled prevalence of PCV in 2802 samples from 38 studies was 43% (95% CI: 2-4%), τ^2^=4.253; p<0.01** (Figure-12). Although some variations are seen among various studies, the heterogeneity is significant; hence, the random effect model was chosen [106-109].

### Prevalence of CSF

**Figure-13:**
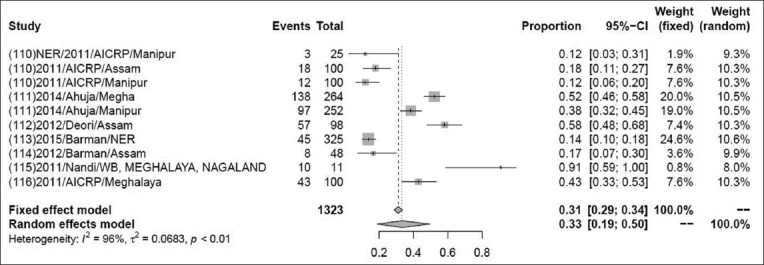
Forest plot of studies on the prevalence of classical swine fever.

The pooled prevalence of CSF in 1323 samples from 11 studies was 31% (95% CI: 2-4%), τ^2^=1.088; p<0.01** (Figure-13). Although some variations are seen among various studies, the heterogeneity is significant; hence, the random effect model was chosen [110-116].

Table-1 provides an insight into the overall meta-analysis summary of 13 livestock diseases.

**Table-1:** Summary of meta-analysis results.

S. No.	Study	Total number of studies	Total number of samples	Number of positive samples	Meta-analysis results						

					Model	Proportion	95% CI	I^2^ (%)	Tau^2^	Relative weightage range (%)	p value
1	Babesiosis	11	1238	73	Random	0.06	(0.03; 0.12)	77	1.07	4.1-10.5	<0.01
2	Bluetongue	11	2762	917	Random	0.28	(0.21; 0.36)	95	0.383	7.5-9.6	<0.01
3	Foot-and-mouth disease	39	41,009	9260	Random	0.21	(0.18; 0.24)	98	0.368	2.2-2.6	0
4	Peste des petits ruminants	23	5221	1192	Random	0.15	(0.09; 0.25)	94	1.61	3.7-4.7	<0.01
5	Porcine cysticercosis	13	4810	314	Random	0.06	(0.04; 0.09)	88	0.753	5.8-8.8	<0.01
6	Theileriosis	8	1468	418	Random	0.26	(0.21; 0.32)	74	0.114	2.5-15.5	<0.01
7	Porcine reproductive and respiratory syndrome	41	91,904	1929	Random	0.01	(0.00; 0.02)	97	3.522	0.8-2.6	<0.01
8	Brucellosis bovine	14	54,056	5190	Random	0.17	(0.08; 0.29)	99	0.065	6.7-7.2	<0.01
9	Brucellosis caprine	2	1164	28	Random	0.02	(0.02; 0.04)	0	0	7.2-92.8	0.44
10	Brucellosis porcine	2	448	68	Random	0.18	(0.00; 0.99)	99	13.36	49.9-50.1	<0.01
11	Brucellosis sheep and goat	4	1129	31	Random	0.03	(0.02; 0.04)	0	0	16.2-32.5	0.59
12	*Porcine circovirus*	38	2808	1345	Random	0.43	(0.28; 0.60)	94	4.253	1.9-2.9	<0.01
13	Classical swine fever	10	1323	431	Random	0.31	(0.18; 0.47)	95	1.088	5.6-11.1	<0.01

CI=Confidence interval

## Discussion

The present study was intended to know the overall prevalence of livestock diseases in NER of India by meta-analyses of reports on the prevalence of the diseases. Large data set is important for projecting country-level prevalence and to identify the severely affected regions and mobilization of resources. Meta-analysis has become the standard for quantitative evidence synthesis, offering a broadly accepted and statistically powerful framework for integrating and adding value to previously published large databases containing raw or partially annotated information. In addition, data mentioned in annual reports of AICRP, annual reports on livestock diseases, State Disease Diagnostic Lab Monthly Progress Report, Progress Report of North East Regional Disease Diagnostic Laboratory, Annual Report of Disease investigation laboratory of NER, Lab maintained data of Directorate of Veterinary and Animal Husbandry Services, and lab reports of Animal Resource Development Department of NER also were taken into account for the calculation of total prevalence.

The present study indicates the pooled prevalence rate of various livestock diseases. As per the present study, the pooled prevalence rate of babesiosis for a sample size of 1238 from 11 studies was 6% (95% CI: 3-12%), and this finding is slightly lower than the previous study, which showed that overall prevalence of babesiosis in cattle was 8.78% conducted in and around Guwahati, the headquarter of Kamrup (Metropolitan) district, and the capital city of Assam [117]. The pooled prevalence rate of bluetongue in 2762 samples from 11 studies was 28% (95% CI: 21-36%), as per the earlier studies, overall 43.07% seroprevalence of bluetongue virus group-specific antibodies was detected in goats and cattle, this was showing higher prevalence rate than the present study [118]. The pooled prevalence rate of foot-and-mouth disease in 41,009 samples from 39 studies was 21% (95% CI: 18-24%), foot-and-mouth disease virus in bovine of Diyala province appeared with the high seroprevalence (25.33%) when 114 out of 450 tested animal sera were positive by 3ABC test to non-structural protein antigens, this was slightly higher than the present study [119]. The pooled prevalence rate of PPR in 5221samples from 23 studies was 15% (95% CI: 9-25%), as per the previous study, it indicates the lower prevalence rate (15.79%) [56]. The pooled prevalence rate of porcine cysticercosis in 4810 samples from 13 studies was 6% (95% CI: 4-9%). The earlier studies indicate that the prevalence rate of porcine cysticercosis was ranged from 5.71% to 14.06%, it was showing variations because the prevalence rate was determined based on the sex, breed, season, etc. [120]. The pooled prevalence rate of theileriosis in 1468 samples from eight studies was 26% (95% CI: 21-32%). This finding is much lower than the previous investigation finding 620 cattle blood samples that were screened using Giemsa’s staining technique. The microscopic examination of blood smears revealed 9.35%, overall prevalence of theileriosis. The highest prevalence was found in the summer season with a prevalence rate of 13.3% which indicates that theileriosis spread more in hot and humid weather (summer season) [121].

The pooled prevalence rate of PRRS in 91,904 samples from 41 studies was 1% (95% CI: 0-2%). The pooled prevalence rate of caprine brucellosis in 1164 samples from two studies was 2% (95% CI: 2-4%). The previous reports suggested that the prevalence rate of caprine brucellosis was 2.30% above 18 months of age, higher than the present study [104]. The pooled prevalence rate of porcine brucellosis in 448 samples from two studies was 33% (95% CI: 0-99%). The pooled prevalence rate of sheep and goat brucellosis in 1129 samples from four studies was 3% (95% CI: 2-4%), which was showing higher prevalence rate than the previous study, it means prevalence of brucellosis was recorded, 1.45% in goat by RBPT, STAT, and I-ELISA [122]. The pooled prevalence of PCV in 2802 samples from 38 studies was found to be 43% (95% CI: 2–4%), while a previous study suggested that the prevalence rate was higher in a total of 1899 serum samples collected and screened using antibody ELISA kits specific for PCV2 showed 79.1% [123]. Pooled prevalence of CSF in 1323 samples from 11 studies was found to be 31% (95% CI: 2–4%) but in an earlier study, prevalence of classical swine fever virus in India was reported to be 37% (95% CI=0.24, 0.51) for a sample size of 6158 which was higher than the present investigation [7].

## Conclusion

There were mixed reports about the prevalence of diseases in animals. Some studies show a larger prevalence while some are less. Meta-analysis is a set of statistical techniques to combine information from various studies to derive an overall and precise estimate of prevalence. The current meta-analysis may represent the precise estimate of the prevalence of diseases when large number of studies show confusing results. Our analysis suggests that the prevalence of various livestock diseases is foot-and-mouth disease (21%), bluetongue (28%), brucellosis in bovine (17%), brucellosis in caprine (2%), brucellosis in porcine (18%), brucellosis in sheep and goat (3%), babesiosis (9%), theileriosis (28%), PRRS (2%), porcine cysticercosis (6%), CSF (31%), PCV (43%), and PPR (15%). This information helps policymakers to take appropriate measures to reduce the disease burden.

## Authors’ Contributions

NNB and SSP conceptualized the aim of the study, designed, planned, supervised the analysis, and corrected the manuscript. KPS, RK, and GD performed all analyses, prepared the graphs, figures, and tables. KMR and CS drafted the manuscript. PD, DPB, and PR provided conceptual support and critically reviewed the manuscript. All authors have read and approved the final manuscript.
